# Effects of Mobilization Within 24 Hours Based on the ICU Mobility Scale in Cancer Patients: A Randomized Controlled Clinical Trial “Mobilization Based on the ICU Mobility Scale”

**DOI:** 10.1002/jso.28142

**Published:** 2025-05-13

**Authors:** Flaviana Santos de Sousa Silva, Giérisson Brenno Borges Lima, Gabriel Santos de Castro e Lima, Denise Carvalho Torres, Michel Monteiro Macedo, Carlos Eduardo Neves Amorim

**Affiliations:** ^1^ Federal University of Maranhão (UFMA) Sao Luís Brazil; ^2^ University Center UniRedentor (UniRedentor) Itaperuna Brazil

**Keywords:** early mobilization, intensive care unit, surgical oncology

## Abstract

**Background and Objective:**

Abdominal cancer surgery leads to loss of functional capacity. The objective was to evaluate the effects of mobilization within 24 h applied to patients with abdominal neoplasms undergoing major surgery.

**Methods:**

A randomized controlled clinical trial was carried out in the Intensive Care Unit. The intervention group performed mobility activities guided by the ICU mobility scale (IMS) in the first 24 h after surgery and the control group performed conventional physiotherapy. Dynamometry was evaluated in the preoperative, 1st POD and postoperative period, as well as the Timed up and go test (TUG).

**Results:**

Patients in the intervention group had greater initial mobility (IMS Scale intervention group: 6.67 ± 0.69; IMS Scale control group: 2.23 ± 0.52; *p* = 0.001). There was greater level of mobility until discharge from the ICU in patients in the intervention group compared to the control group (IMS at discharge from the ICU in the intervention group: 8.53 ± 0.33; IMS at discharge from the ICU in the control group: 3 ± 0.64). Both groups showed worsening in the TUG test, but it was significant only in the control group.

**Conclusion:**

Early mobilization in patients with abdominal neoplasms undergoing major surgery proved to be effective in maintaining mobility and functional markers.

## 1 Introduction

Major abdominal surgery can lead to loss of functional capacity during hospitalization. Even in the absence of complications, the postoperative period is associated with a 20%–40% reduction in physiological and functional capacity, especially in elderly patients with comorbidities, which may delay a return to preoperative function for several months [1, 2, 3]. Low preoperative physical performance increases the risk of the number of postoperative complications [2, 3].

Admission to an intensive care unit (ICU) following surgical procedures may be required. Studies show that prolonged stays in this environment are associated with long‐term impairments, such as ICU‐acquired muscle weakness, which can negatively impact patients’ quality of life [1, 4]. Early mobilization is recommended for patients undergoing elective abdominal surgery and is considered a therapeutic strategy to improve respiratory function and prevent postoperative complications [5, 6]. The use of systematic mobilization protocols is one of the strategies the multidisciplinary team employs to facilitate mobility gains throughout the patient's hospitalization. It is crucial to determine the optimal level of mobilization at different time points in the ICU [7, 8, 9].

Tools are available to assess the physical and functional status of patients admitted to intensive care units and monitor their performance during their stay. The ICU Mobility Scale (IMS) is a tool that has been widely used in ICUs to evaluate, guide clinical decisions, and track the progression of activities related to the mobilization of critically ill patients. The IMS scores range from 0 to 10, representing levels of mobility from low to high [10, 11].

Early mobilization is considered a key element in postoperative recovery for patients undergoing abdominal surgery and is strongly recommended by the Enhanced Recovery After Surgery Society (ERAS) perioperative care guidelines. However, the level of evidence supporting its use remains low, and adherence to this intervention is still suboptimal [12, 13].

Our hypothesis is that cancer patients undergoing major abdominal surgery who receive individualized early mobilization interventions guided by their mobility level will show better functional and clinical outcomes than those subjected to conventional mobilization strategies. The objective of this study was to evaluate the effects of mobilization within 24 h applied to patients with abdominal neoplasms undergoing major surgery.

## 2 Materials and Methods

### 2.1 Study Design and Setting

This was a randomized controlled clinical trial conducted from December 2021 to August 2022 in São Luís, Maranhão, in the ICU of a cancer treatment referral hospital.

### 2.2 Ethics and Informed Consent

The study was approved by the Ethics and Research Committee of the University Hospital of the Federal University of Maranhão, under approval number 4.980.296, following the ethical principles established by Resolution No. 466/12 of the National Health Council and its complementary guidelines, as well the Declaration of Helsinki [14]. Data collection commenced after participants agreed to sign the informed consent form.

### 2.3 Sample Size

We conducted a post hoc power analysis using G*Power 3.1.9.7 to evaluate the statistical power of our study for the ANOVA: Repeated measures, within‐between interaction test. The analysis used the following input parameters: an effect size (f) of 0.25, an alpha error probability (α) of 0.05, a total sample size of 30 participants, 2 groups, and 2 repeated measurements. The correlation among repeated measures was set to 0.5, and the nonsphericity correction factor (ε) was 1.

The results indicated a noncentrality parameter (λ) of 7.5, a critical F value of 4.19597, and degrees of freedom for the numerator (df = 1) and denominator (df = 28). The computed post hoc power (1‐β error probability) was 0.7529 (75.29%). This suggests that the study had a 75.29% chance of detecting a true effect of the specified size under the given conditions.

### 2.4 Patients

Patients eligible for inclusion were those undergoing major abdominal surgery who had medical clearance for early mobilization according to the study protocol, aged ≥ 18 years, with Barthel Index scores of 80–100 within 2 weeks before hospitalization, Eastern Cooperative Oncology Group (ECOG) performance status ≤ 2 and Karnofsky performance status ≥ 50%. Patients admitted postoperatively were either on spontaneous ventilation or required less than 48 h of mechanical ventilation.

Exclusion criteria included patients with neurological sequelae, inability to follow commands or perform tests, myocardial infarction within 30 days before surgery, unstable angina, uncontrolled cardiac arrhythmias, severe valvular heart disease, congestive heart failure classified as New York Heart Association (NYHA) Class III or IV, venous thromboembolism, pericarditis or myocarditis, acute endocarditis, acute aortic dissection, thyrotoxicosis, inability to ambulate independently, inability to exercise, presence of bone metastases, active infection, musculoskeletal and neurological conditions preventing adherence to the protocol, palliative procedures, or refusal to provide consent.

During the intervention, only the principal investigator knew the patients’ group assignment (intervention vs. control). Researchers independent of those managing the control group (which followed the hospital's conventional treatment protocol) conducted the mobilization for the intervention group. Independent researchers also collected and analyzed data, with the intervention researchers tabulating the data and a separate researcher conducting the statistical analysis.

All patients included in the study were analyzed statistically, even if data were incomplete due to loss to follow‐up or death, to adhere to this study's intention‐to‐treat principle.

### 2.5 Interventions

Demographic and clinical data were collected at three time points: hospital admission, within the first 24 h of ICU admission, and hospital discharge, using medical records or interviews. Subsequently, three scales were applied to assess the patient's degree of independence and prior performance status: the Barthel Index, the ECOG performance status, and the Karnofsky performance scale.

### 2.6 Handgrip Strength

Patients were evaluated at hospital admission (ADM), on the first postoperative day (POD 1), and at hospital discharge (post). They were given at least 6 s to generate a peak maximum force, with a 60‐s rest interval between each test, following the protocols outlined in the referenced studies [15, 16, 17]. The highest score recorded over three attempts was used for analysis.

### 2.7 Timed Up and Go Test

This test was performed at hospital admission and discharge. Participants were instructed to stand up from a chair without using their arms, walk a distance of 3 m as quickly as possible, turn around, and sit back down. A cone was used to mark the distance. The participant was instructed to “go” to begin the test, and the time was recorded from the verbal command until the moment they fully rested their back against the chair [18, 19].

#### 2.7.1 Intervention Group

Patients in the intervention group were approached by the principal investigator after their admission to the ICU and were mobilized out of bed within 24 h, as early as possible. An early and progressive mobilization program was applied according to the ICU Mobility Scale (IMS).

Following the evaluation, the principal investigator encouraged the patients to reach the highest possible level of mobility during the first session. The IMS score was recorded after the patient achieved their maximum mobility level. Initially, patients were positioned sitting up in bed, and if they showed no signs of intolerance, they progressed to sitting at the bedside, followed by standing, stationary marching, and ambulation. Upon returning to the ICU, they were placed in a sitting position in a chair for at least 1 h. The distance walked was measured using markers placed on the ICU and ward corridors floor. Interventions were carried out twice a day (morning and afternoon).

Exercises were halted if the patient experienced intolerance, such as pain (numerical pain scale > 5 or pain preventing mobilization due to refusal), mean arterial pressure < 65 or > 120 mmHg, heart rate > 120 bpm, respiratory rate > 35 breaths per minute, dyspnea causing refusal to continue, or symptoms of imminent syncope.

The patient was encouraged to advance their mobility level during each subsequent session based on their previous IMS score.

#### 2.7.2 Control Group

Patients in the control group received standard care provided by the unit's physiotherapists, including bed positioning, active bed exercises, bed‐to‐chair transfers, standing, and ambulation. However, these practices were not guided by any mobility scale, and the timing of the first mobilization out of bed after surgery was not standardized; it was left to the discretion of the healthcare professionals.

There were independent researchers in each stage of the research: one responsible for collecting data in the preoperative period, on the 1st postoperative day and at hospital discharge, and this researcher was unaware of which patients were in the control group and which were in the intervention group. Another researcher was responsible only for carrying out early mobilization interventions in the ICU, independent professionals to take over the intervention of the control group (from the hospital staff) and an independent researcher for the statistical analyses.

### 2.8 Data Analysis

Descriptive characteristics are presented as mean, standard deviation, frequency, and proportion. The Shapiro–Wilk and Mauchly tests assessed data normality and homoscedasticity, respectively. Independent and paired *t*‐tests were used to compare variables between groups and time points. Categorical variables were compared using the Chi‐square test, while continuous variables were analyzed using two‐way ANOVA (2 × 2, group vs. time). For handgrip strength comparison, repeated‐measures ANOVA was applied for the group (treated and untreated) and time (hospital admission, first postoperative day, and hospital discharge). When sphericity was not met, the Geisser‐Greenhouse correction was applied. Results were considered significant at *p* < 0.05, and all statistical analyses were performed using SPSS 20.0.

## 3 Results

**Figure 1:**
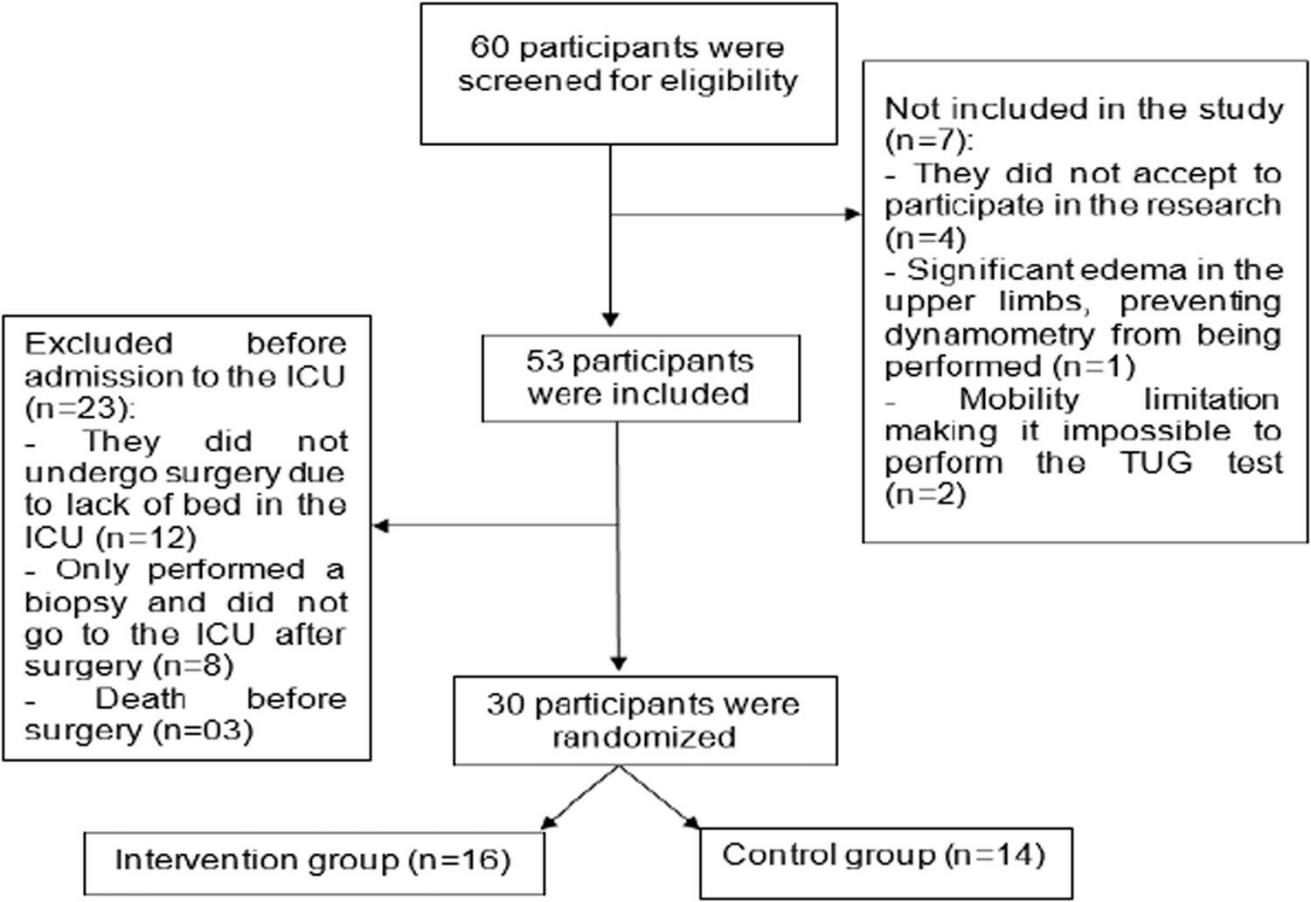
Sample composition. ICU, intensive care unit; MMSS, upper limbs; TUG, timed up and go.

The final sample consisted of 30 patients, 14 in the control and 16 in the intervention group (Figure 1). The groups had similar characteristics in terms of sex (50%) and an average age of 56.7 ± 14 years. Demographic and clinical characteristics are detailed in Table 1.

**Table 1:** Preoperative demographic, clinical, and functional characteristics.

Variables	Intervention (*n* = 16)	Control (*n* = 14)	Total (*n* = 30)	*p*
Sex				0.638
Male	9 (56.25%)	6 (42.85%)	15 (50%)	
Female	7 (43.75%)	8 (57.14%)	13 (50%)	
Age (years)	56.27 ± 15.87	55.85 ± 12.56	56.7 ± 14.0	0.939
Comorbidities				0.270
HAS	1 (6.7%)	3 (23.1%)	4 (14.3%)	
HAS + DM	2 (13.3%)	0	2 (7.1%)	
HAS + DM + obesity	1 (6.7%)	0	1 (3.6%)	
Lifestyle habits				0.208
Smoking	0 (0%)	2 (15.4%)	2 (7.1%)	
Alcohol use	1 (6.7%)	3 (23.1%)	4 (14.3%)	
Smoking + alcohol use	3 (20%)	2 (15.4%)	4 (17.9%)	
BMI (kg/m^2)^	23.85 ± 4.87	22 ± 6.86		0.420
Barthel index	100	100		0.911
KPS	94.67 (± 5.16)	90 (± 7.07)		0.059
ECOG	0.20 (0.41)	0.23 (0.43)		0.850

Abbreviations: BMI, body mass index; DM, diabetes mellitus; ECOG, Eastern Cooperative Oncology Group; HAS, systemic arterial hypertension; KPS, Karnofsky performance status.

The groups had comparable baseline characteristics regarding their ability to perform daily activities before hospital admission, as assessed by the Barthel Index, Karnofsky Performance Status (KPS), and Eastern Cooperative Oncology Group (ECOG) scales (Table 1). Gastric adenocarcinoma was the most frequent tumor in both groups (46.66%). Consequently, gastrectomy was the most commonly performed surgery (46.66%), and 9 patients (32.2%) underwent adjuvant treatments in addition to surgery.

The SAPS 3 prognostic score indicated a low risk of mortality for both groups. Surgical data are also detailed in Table 2.

**Table 2:** Characteristics related to underlying neoplasms, type of surgery performed, prior treatments, type of anesthesia, and surgical risk.

Variables	Intervention (*n* = 16)	Control (*n* = 14)	Total (*n* = 30)		*p*	
Neoplasms						0.115
Gastric adenocarcinoma	7 (43.75%)	7 (50%)	14 (46.66%)			
Colon adenocarcinoma	3 (18.75%)	1 (7.14%)	4 (13.33%)			
Rectal adenocarcinoma	0 (0%)	3 (21.42%)	3 (10%)			
Pancreatic adenocarcinoma	0 (0%)	2 (14.28%)	2 (6.66%)			
Anal canal adenocarcinoma	0 (0%)	1 (7.14%)	1 (3.33%)			
Liposarcoma	1 (6.25%)	0 (0%)	1 (3.33%)			
Gastric adenocarcinoma with liver metastasis	1 (6.25%)	0 (0%)	1 (3.33%)			
Colon adenocarcinoma with liver metastasis	2 (12.5%)	0 (0%)	2 (6.66%)			
Rectal adenocarcinoma with liver metastasis	2 (12.5%)	0 (0%)	2 (6.66%)			
Surgeries					0.44	
Gastrectomy	7 (43.75%)	7 (50%)		14 (46.66%)		
Colectomy	3 (18.75%)	1 (7.14%)		4 (13.33%)		
Rectosigmoidectomy	3 (18.75%)	3 (21.42%)		6 (20%)		
Hepatectomy	1 (6.25%)	0 (0%)		1 (3.33%)		
Duodenopancreatectomy	0 (0%)	2 (14.28%)		2 (6.66%)		
Abdominoperineal amputation	0 (0%)	1 (7.14%)		1 (3.33%)		
Liposarcoma resection	1 (6.25%)	0 (0%)		1 (3.33%)		
Gastroenterostomy	1 (6.25%)	0 (0%)		1 (3.33%)		
Previous treatments					0.423	
None	10 (62.5%)	11 (78.57%)	21 (70%)			
Chemotherapy	4 (25%)	1 (7.14%)	5 (16.66%)			
Radiotherapy	0 (0%)	0 (0%)	0 (0%)			
Chemotherapy and radiotherapy	2 (12.5%)	2 (14.28%)	4 (13.33%)			
Type of anesthesia					0.263	
General	3 (18.75%)	1 (7.14%)	4 (13.33%)			
General + spinal	8 (50%)	6 (42.85%)	14 (46.66%)			
General + peridural	4 (25%)	7 (50%)	11 (36.66%)			
Spinal + peridural	1 (6.25%)	0 (0%)	1 (3.33%)			
Surgical risk					0.630	
Low	14 (87.5%)	13 (92.85%)	27 (90%)			
Moderate	2 (12.5%)	1 (7.14%)	3 (10%)			
Surgery duration (minutes)	157.07 (± 22.36)	168.85 (± 22.36)			0.670	
Intraoperative fluids (mL)	4000 ± 1933.6	3884.3 ± 1914.4			0.870	
SAPS 3	36.33 (± 3.14)	37 (± 2.62)			0.874	
ICU stay duration (days)	2.46 (± 1.64)	3.07 (± 1.70)			0.34	

Abbreviations: ICU, intensive care unit; SAPS 3, simplified acute physiology score.

The patients in the intervention group received their first treatment within 24 h of ICU admission (intervention group: 20.07 ± 0.81; control group: 34.31 ± 3.69; *p* = 0.001), and the total time spent on the first mobilization activity, including the time the patient remained seated in the chair and/or ambulating, was also more significant in the intervention group (intervention group: 104.67 ± 24.16 min; control group: 26.15 ± 9.09 min; *p* = 0.008) (Table 3).

**Table 3:** Differences between groups related to mobilization in the ICU.

Variable	Intervention (*n* = 16)	Control (*n* = 14)	*p*
Time to 1st ICU treatment (hours)	20.07 (± 0.81)	34.31 (± 3.69)	0.001*
Total time of mobilization activity on 1st POD (minutes)	104.67 (± 24.16)	26.15 (± 9.09)	0.008*
Time to first out‐of‐bed mobilization (hours)	21.9 (± 1.67)	66.46 (± 10.92)	0.002*
Initial IMS	6.67 (± 0.69)	2.23 (± 0.52)	0.002*
0	0	0	
1–2	1 (6.25%)	9 (64.29%)	
3	2 (12.5%)	2 (14.28%)	
4–6	4 (25%)	2 (14.28%)	
7	0	1 (7.15%)	
8–10	9 (56.25%)	0	
IMS at ICU discharge	8.53 (± 0.33)	3 (± 0.64)	0.001*
0	0	0	
1–2	0	7 (50%)	
3	0	3 (21.43%)	
4–6	2 (12.5%)	2 (14.28%)	
7	0	2 (14.28%)	
8–10	14 (87.5%)	0	

Abbreviations: ICU, intensive care unit; IMS, ICU mobility scale; POD, postoperative day.

The time to first mobilization out of bed was significantly shorter in the intervention group compared to the control group. While patients in the intervention group took an average of 21.9 (± 1.67) hours to get out of bed for the first time, the control group took an average of 66.46 (± 10.92) hours, approximately 3 days (Table 3).

Table 3 describes the mobility levels during the first treatment and at ICU discharge according to the IMS score in each group. We observed that, of the 16 patients in the intervention group, 13 (81.25%) achieved an IMS score of 4–10 during the first treatment. Of these, 9 (56.25%) reached an IMS score between 8 and 10, meaning they could ambulate with assistance from only one person, using a mobility aid, or independently. Only three patients had an IMS score of 1–3 due to pain complaints that limited their mobility progression, but these patients were able to get out of bed by the second treatment.

The progression of mobility level until ICU discharge was significantly greater in the intervention group compared to the control group (IMS at ICU discharge for the intervention group: 8.53 (± 0.33); IMS at ICU discharge for the control group: 3 (± 0.64); *p* = 0.001).

Only 2 patients in the control group (14.28%) reached an IMS score of 7 at ICU discharge, and none achieved an IMS score between 8 and 10. In contrast, all 16 patients (100%) in the intervention group reached an IMS score between 4 and 10, with 14 (87.5%) attaining an IMS score between 8 and 10.

All characteristics related to mobilization between the groups are shown in Table 3.

There was no significant difference in handgrip strength at the three‐time points or between the groups (Table 4).

**Table 4:** Handgrip strength at hospital admission, 1st postoperative day, and hospital discharge.

	Handgrip strength (KGF)	Handgrip strength (KGF)	
Evaluation phases	Intervention (*n* = 16)	Control (*n* = 14)	*p*
Preoperative	31.73 (± 10.82)	32.08 (± 8.47)	0.92
1st POD	29.87 (± 11.69)	27.15 (± 8.16)	0.49
Hospital discharge	32.29 (± 11.31)	27.23 (± 9.61)	0.22

Abbreviations: KGF, kilogram‐forcel; POD, postoperative day.

**Graph 1:**
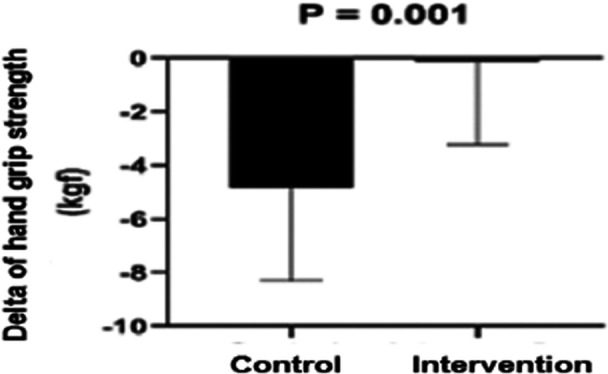
The graphs represent handgrip strength at preoperative, 1st postoperative day, and hospital discharge. Significance level: *p* < 0.05, using two‐way ANOVA. KGF, kilogram‐force.

Although no differences were found in the raw handgrip strength values, the delta variation between admission and hospital discharge in the control group showed a more significant strength decline than in the intervention group, as shown in Graph 1.

**Graph 2:**
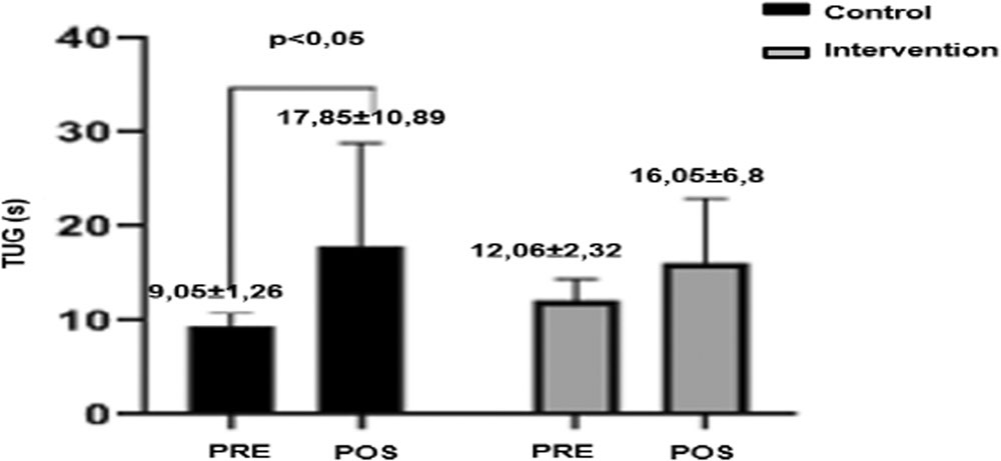
Timed Up and Go test at hospital admission and discharge. Significance level: *p* < 0.05, using repeated‐measures ANOVA. TUG, timed up and go test.

Both groups showed an increase in the time required to perform the TUG test; however, it was observed that the control group performed the test in less time at admission compared to the intervention group (9.05 ± 1.26 vs. 12.06 ± 2.32, respectively), and only the control group showed a deterioration in performance for the TUG test, requiring more time to complete the test at hospital discharge (Graph 2). The control group patients had a more extended hospital stay than the intervention group, although this difference was not statistically significant (*p* = 0.1).

## 4 Discussion

Mobilization out of bed within 24 h is tolerated and recommended according to enhanced recovery programs after abdominal surgery. Although many studies have demonstrated the benefits of using mobility scales in the ICU to guide mobilization goals, their specific use for surgical patients with abdominal neoplasms is rare. In the present study, we adopted the IMS to guide mobilization goals in patients in the intervention group [11, 13].

Patients in the intervention group could begin their first treatment, on average, 14 h earlier than the control group, and they achieved higher levels of mobility within 24 h postsurgery.

Koyuncu et al. [13], following an early mobilization protocol based on ERAS guidelines, mobilized patients out of bed even earlier than in this study. In their intervention group, patients were mobilized at 6.22 ± 1.95 h, while the control group was at 12.21 ± 3.76 h (*p* < 0.05) after major abdominal surgeries (pancreatic, gastrointestinal, and colorectal cancers). However, the control group patients were unable to achieve all mobilization goals within the first 24 h postsurgery, and the authors did not use any tool to assess patient mobility [13]. This highlights the importance of using mobilization protocols considering individual characteristics based on each patient's mobility level.

As in the present study, other authors have also used the IMS to guide mobilization goals. Hodgson et al. [11], in a randomized trial conducted in five ICUs with medical, surgical, and trauma beds in Australia and New Zealand, adopted the IMS to guide mobilization goals with a protocol that included active functional activities and higher levels of activity and mean IMS. Patients whose mobility was guided by IMS‐based goals achieved higher mobility levels at ICU discharge than the control group [11]. However, the study had a more heterogeneous sample than ours, restricted to surgical patients with abdominal neoplasms.

Another important finding relates to the duration of the first mobilization activity, which was also longer in the intervention group (intervention group: 104.67 ± 24.16 min; control group: 26.15 ± 9.09 min; *p* = 0.008). We believe that the use of guiding instruments and an intention in early mobilization is what brought greater efficiency to mobilization. Demanding a longer intervention time.

The progression of mobility level until ICU discharge in the intervention group was significantly greater than in the control group (IMS at ICU discharge for the intervention group: 8.53 (± 0.33); IMS at ICU discharge for the control group: 3 (± 0.64); *p* = 0.001).

Almeida et al. [1] evaluated the effect of an early mobilization protocol in a sample of 108 patients postmajor abdominal oncologic surgeries. The intervention group, subjected to early mobilization, performed more exercises and showed better functional capacity at hospital discharge, which is consistent with our findings. The early mobilization program consisted of a set of exercises initiated on the first postoperative day [1].

In the present study, no significant difference was found in the raw values of handgrip strength across the three time points; however, in the delta variation between hospital admission and discharge, the control group showed a more significant decline in strength, indicating the possibility of strength loss during hospitalization.

Lachmann et al. [20] conducted a prospective observational study assessing clinically measurable peripheral weakness and functional decline. All patients showed a significant decrease in handgrip strength on the first postoperative day, at ICU discharge, and at hospital discharge. In their study, no early mobilization or other ERAS‐guided interventions were used, similar to our study, emphasizing the importance of guiding mobilization using scales that direct the level of mobility [20].

Both groups showed increased time required to perform the TUG test; however, this increase was significant only in the control group. The TUG test is known to assess functional capacity. In addition to being related to fall risk in the elderly, it can also be associated with complications and mortality up to 1 year postsurgery. However, this correlation was not observed in the present study. Hendriks et al. [21] conducted a prospective observational cohort study evaluating 5‐year mortality in elderly patients with solid tumors who underwent surgery, correlating it with prior physical performance. Elderly patients with impaired TUG, defined as a mean time > 12 s, had worse outcomes with increased mortality.

In a multicenter prospective cohort of 263 patients (≥ 70 years) undergoing surgery for solid tumors, individuals with a high TUG (> 20 s) had a 3.43 times higher risk of developing significant complications within 30 days postoperatively compared to patients with normal TUG (95% CI = 1.13–10.36; *p* = 0.03), and a 4.21 times higher risk of prolonged hospitalization [22].

Regarding the length of hospital stay, this study did not find a significant difference between the groups. Different findings were observed in a meta‐analysis by Bond‐Smith et al. [23], which found that hospital stays were significantly shorter in interventions with enhanced recovery protocols, including mobilization within the first 48 h and postoperative enteral nutrition, compared to the standard treatment group. The reduction in hospital stay also seems to be influenced by the combination of early mobilization and other multimodal measures adopted in the preoperative, intraoperative, and postoperative periods, as recommended by the ERAS protocol [24].

Some limitations can be mentioned. Because the study was not conducted in an exclusively surgical ICU, bed availability was reduced, leading to the exclusion of many patients. Some patients in both groups remained on a zero diet for the first 48 h after surgery, receiving only glucose saline, which may have influenced their disposition to perform the proposed activities. Nevertheless, the intervention group was able to perform the activities, with pre‐intervention blood glucose monitoring as a safety measure.

The COVID‐19 pandemic also influenced the study, as although surgeries had resumed, not all were being performed. Therefore, further research with more patients is needed, as well as the possibility of using protocols in specific types of surgery to make the sample more homogeneous.

The study highlights potential positive clinical impacts, such as maintenance of strength and functionality in the intervention group during hospitalization, which may translate into a better functional prognosis, allowing these individuals to remain active after discharge and reducing the risks of readmissions or complications caused by hypomobility. However, these benefits after hospital discharge still require further investigation.

Finally, new studies are suggested to confirm the benefits of early mobilization in surgical cancer patients and its impact on their long‐term quality of life and survival.

## 5 Conclusions

Mobilization within the first 24 h in patients with abdominal neoplasms undergoing major surgery proved effective and improved mobility markers during the ICU stay.

## Synopsis

This study is a prospective, randomized clinical trial in which patients with abdominal cancer undergoing major surgery were randomized to undergo mobilization within 24 h after surgery or non‐standardized mobilization. Greater mobility achieved within 24 h after surgery appears to influence less functional loss.

## Data Availability

The authors have nothing to report.
